# Pterostilbene Attenuates Subarachnoid Hemorrhage-Induced Brain Injury through the SIRT1-Dependent Nrf2 Signaling Pathway

**DOI:** 10.1155/2022/3550204

**Published:** 2022-11-30

**Authors:** Zihuan Zhang, Jincheng Fang, Jiawang Zhou, Fei Ding, Gang Zhou, Xintong Zhao, Zong Zhuang, Yue Lu

**Affiliations:** ^1^The Translational Research Institute for Neurological Disorders of Wannan Medical College, Department of Neurosurgery, The First Affiliated Hospital of Wannan Medical College (Yijishan Hospital of Wannan Medical College), Wuhu 241001, China; ^2^Department of Neurosurgery, Nanjing Drum Tower Hospital, The Affiliated Hospital of Nanjing University Medical School, Nanjing, 210008 Jiangsu, China

## Abstract

Neuroinflammatory injury, oxidative insults, and neuronal apoptosis are major causes of poor outcomes after subarachnoid hemorrhage (SAH). Pterostilbene (PTE), an analog of resveratrol, has been verified as a potent sirtuin 1 (SIRT1) activator. However, the beneficial actions of PTE on SAH-induced brain injury and whether PTE regulates SIRT1 signaling after SAH remain unknown. We first evaluated the dose-response influence of PTE on early brain impairment after SAH. In addition, EX527 was administered to suppress SIRT1 signaling. The results revealed that PTE significantly attenuated microglia activation, oxidative insults, neuronal damage, and early neurological deterioration. Mechanistically, PTE effectively enhanced SIRT1 expression and promoted nuclear factor-erythroid 2-related factor 2 (Nrf2) accumulation in nuclei. Furthermore, EX527 pretreatment distinctly repressed PTE-induced SIRT1 and Nrf2 activation and deteriorated these beneficial outcomes. In all, our study provides the evidence that PTE protects against SAH insults by activating SIRT1-dependent Nrf2 signaling pathway. PTE might be a therapeutic alternative for SAH.

## 1. Introduction

Subarachnoid hemorrhage (SAH) remains a catastrophic neurovascular disorder [1]. Early brain injury (EBI) is one of the main causes for poor prognosis after SAH. Thus, therapeutic options for targeting EBI might improve long-term neurological outcomes in SAH patients. Mounting evidence indicates that severe oxidative insults, imbalanced inflammatory injury, and cell apoptosis play vital roles in SAH-induced early brain damage [2–4]. However, effective pharmacotherapies for reversing the progression of EBI remain lacking. Thus, identifying new drugs for SAH patients is urgently needed.

Resveratrol (RSV) is extracted from pines and blueberry. It has been demonstrated that RSV is a powerful sirtuin1 (SIRT1) activator and could effectively protect against SAH-induced brain injury by suppressing oxidative damage, neuroinflammation, and neuronal death [5, 6]. Pterostilbene (PTE), an analog of RSV, has also been verified as a potent SIRT1 activator [7]. In addition, PTE has better oral bioavailability and higher bioactivity than RSV. Accumulating evidence has demonstrated that PTE exhibits different protective activities [8–11]. In neurological diseases, PTE serves cerebroprotective effects against Alzheimer's disease, cerebral ischemia, and aging-related diseases. However, the role of PTE after SAH and the precise mechanisms remain unknown. Therefore, we aimed to illuminate whether PTE ameliorated brain damage following SAH and the effects of PTE on SIRT1-mediated signaling.

## 2. Methods and Materials

### 2.1. Animal Model

All experiments were implemented by the Animal Ethics Review Committee of Yijishan Hospital (approved number: 2019A0423) [12, 13]. Health male SD rats (250–300 g) were conducted for model construction. We used a prechiasmatic cistern injection model of SAH in male rats [14, 15]. According to previous protocols, rats were firstly anaesthetized with avertin. Then, 0.35 ml arterial blood (extract from the femoral artery) was slowly infused into the prechiasmatic cistern. Sham-treated rats underwent similar procedures, with the injection of physiologic saline. Buprenorphine (0.1 mg/kg) was used to reduce analgesia after model construction.

### 2.2. Experiment Groups

In this study, 159 rats were used. Nineteen rats died after model construction, and 8 rats were excluded. Animals were randomized into experimental groups. In the first set experiment, rats were randomized into sham group (vehicle), sham + PTE group (10 mg/kg), SAH group, SAH + vehicle group, SAH + PTE group (5 mg/kg), SAH + PTE group (10 mg/kg), and SAH + PTE group (20 mg/kg). In the second set experiment, rats were randomized into sham group (vehicle), SAH group (vehicle), SAH + PTE group (10 mg/kg), and SAH + PTE + EX527 group (10 mg/kg PTE). Animals groups and mortality rates were shown in Supplementary Table 1.

### 2.3. Drug Treatment

PTE was resolved in dimethylsulfoxide (DMSO) with physiological saline. Three doses of PTE (5, 10, or 20 mg/kg) or vehicle was injected intraperitoneally at 2 h and 12 h after model construction. The doses of PTE were chosen based on previous researches [7, 16]. EX527 (dissolved in 1% DMSO, 15 mg/kg) was injected intraperitoneally for 3 days before model construction [17].

### 2.4. Neurobehavior Function

The modified Garcia score was employed to assess behavior deficits in different groups [18]. Rotarod performance was conducted as previously reported [19, 20]. The latency to fall from rotarod was recorded. The detailed procedures were conducted according to previous studies [21]. Neurological function was recorded by two investigators blinded to the grouping.

### 2.5. Biochemical Evaluation

The endogenous antioxidants and the lipid peroxidation biomarker malondialdehyde (MDA) were determined via using different commercial assay kits (Beyotime Biotechnology). The hydrogen peroxide (H_2_O_2_) expression was detected via using a commercial H_2_O_2_ assay kit (Abcam).

### 2.6. Western Blotting

Western blotting was conducted according to previous studies [22]. First, RIPA lysis buffer was used to extract protein samples in brain tissues. Protein concentrations in each sample were measured before western blot analysis. Equal protein extracts in different groups were moved to polyvinylidene difluoride membranes. After blocking with 5% nonfat milk, primary antibodies against SIRT1 (SC-15404), nuclear factor-erythroid 2-related factor 2 (Nrf2, ab31163), heme oxygenase-1 (HO-1, ab13243), SOD-1 (ab51254), NAD(P)H dehydrogenase quinone 1 (NQO-1, ab34173), Histone H3 (BS1660), and *β*-actin (AP0060) were employed to incubate with these membranes. Then, bands were hatched with the species-corresponding secondary antibodies. Band intensities were analyzed by ImageJ.

### 2.7. ELISA Assay

After sample preparation, the expressions of proinflammatory cytokines were determined using the respective commercial kits (Multi Sciences. China). The concrete steps were performed in consistent with the manufacturer's instructions.

### 2.8. Immunofluorescence Staining

In line with previous studies [23], frozen sections of brain tissue were first fixed in paraformaldehyde (4%). Following incubated with Triton X-100 (0.1%), sections were then blocked with 1% bovine serum albumin. The samples were then hatched with primary antibodies against Iba-1 (1 : 100), Nrf2 (1 : 100), and SIRT1 (1: 50) overnight followed by proper secondary antibodies. After several time washing with phosphate buffered saline, sections were then stained by DAPI solution. Immunofluorescence quantification was recorded by using ImageJ.

### 2.9. TUNEL Staining

TUNEL staining was according to the manufacturer's instructions. First, frozen sections of brain tissue were fixed in paraformaldehyde (4%), treated with Triton X-100 (0.1%), and blocked with 1% bovine serum albumin. After that, brain slices were hatched with primary antibodies against NeuN (1: 200) followed by incubation with TUNEL reaction mixture. The number of TUNEL-positive cells was recorded by ImageJ.

### 2.10. Statistical Analysis

Shapiro–Wilk's test was applied for data normality. Experimental data were normally distributed and were indicated as mean ± SD. Software GraphPad Prism 8.02 was used for data analysis. Neurological behavior scores were evaluated by two-way ANOVA. One-way ANOVA with Bonferroni post hoc test was employed to determine where those differences occurred. The difference *P* < 0.05 was considered significant.

## 3. Results

### 3.1. PTE Dose-Dependently Reduced Oxidative Damage after SAH

**Figure 1:**
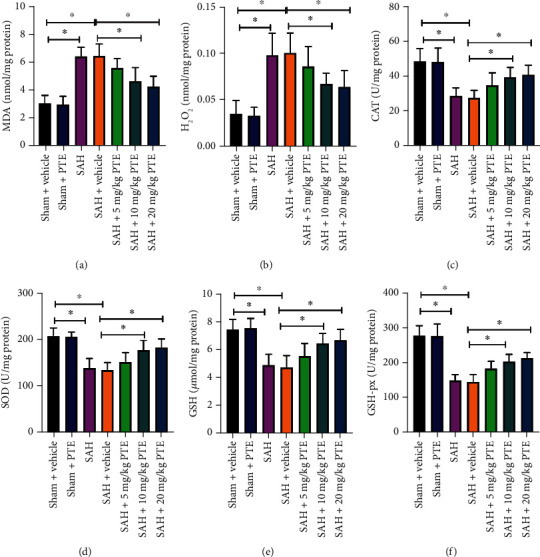
Dose-response effects of PTE on SAH-triggered oxidative insults. PTE treatment resulted in reduced MDA (a), and H_2_O_2_ (b) levels, and better CAT (c), SOD (d), GSH (e), and GSH-Px (f) activities (*n* = 6/groups). One-way ANOVA with Bonferroni post hoc test was employed. Error bars indicated the SD. ^∗^*P* < 0.05.

Oxidative damage is closely associated with SAH-induced early brain insults. As shown, PTE treatment (10 and 20 mg/kg) distinctly suppressed SAH-induced increase in MDA (*P* = 0.002 and *P* < 0.001, respectively) and reactive oxygen species (ROS) overproduction (*P* = 0.034 and *P* = 0.0161, respectively). The decreased antioxidants including catalase (CAT) (*P* = 0.03 and *P* = 0.011, respectively), superoxide dismutase (SOD) (*P* = 0.005 and *P* = 0.0012, respectively), glutathione (GSH) (*P* = 0.009 and *P* = 0.0021, respectively), and glutathione peroxidase (GSH-px) (*P* = 0.0025 and *P* < 0.001, respectively) were also restored after PTE treatment (10 and 20 mg/kg) (Figures 1(a)–1(f)). However, PTE administration at dose of 5 mg/kg did not statistically ameliorate oxidative insults in the early period after SAH (*P* > 0.05). In addition, no statistical differences in oxidative insults-related parameters between 10 mg/kg PTE-treated and 20 mg/kg PTE-treated groups were observed. Therefore, a dose of 10 mg/kg PTE treatment was used for the second set of experiment.

### 3.2. PTE Dose-Dependently Reduced Neuroinflammation after SAH

**Figure 2:**
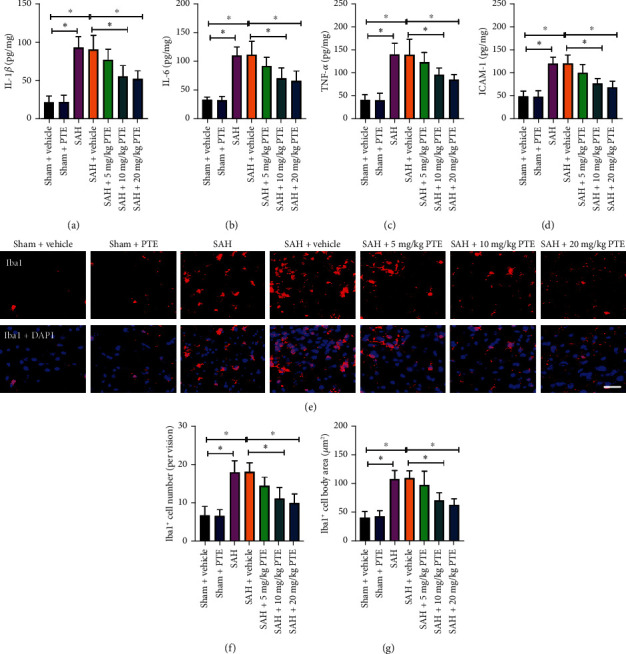
Influence of PTE on neuroinflammation following SAH. PTE treatment resulted in reduced IL-1*β* (a), IL-6 (b), TNF-*α* (c), and ICAM-1 (d) levels (*n* = 6/groups). Representative Iba1 images (e) and quantitative analyses of the density of Iba1^+^ cells (f) and Iba^+^ cell body size (g) in different groups (*n* = 6/groups). One-way ANOVA with Bonferroni post hoc test was employed. Scale bar, 50 *μ*m. Error bars indicated the SD. ^∗^*P* < 0.05.

The disturbed inflammatory response further aggravates brain damage. In this experiment, it revealed that SAH insults evidently induced a variety of proinflammatory cytokines release, which could be decreased by 10 mg/kg (*P* < 0.001 for IL-1*β*, *P* < 0.001 for IL-6, *P* = 0.0089 for TNF-*α*, and *P* < 0.001 for ICAM-1) or 20 mg/kg (*P* < 0.001 for IL-1*β*, *P* < 0.001 for IL-6, *P* < 0.001 for TNF-*α*, and *P* < 0.001 for ICAM-1) PTE treatment (Figures 2(a)–2(d)). The brain resident microglia could release proinflammatory mediators and contribute to the disturbed inflammatory injury after SAH. Our data further indicated that PTE administration dose-dependently inhibited SAH-induced microglia activation, including the Iba1-positive number (*P* < 0.001) and microglia cell body area (*P* < 0.001) (Figures 2(e)–2(g)). These results suggested that PTE could protect against neuroinflammation after SAH.

### 3.3. PTE Improved Functional Recovery and Reduced Neuronal Apoptosis after SAH

**Figure 3:**
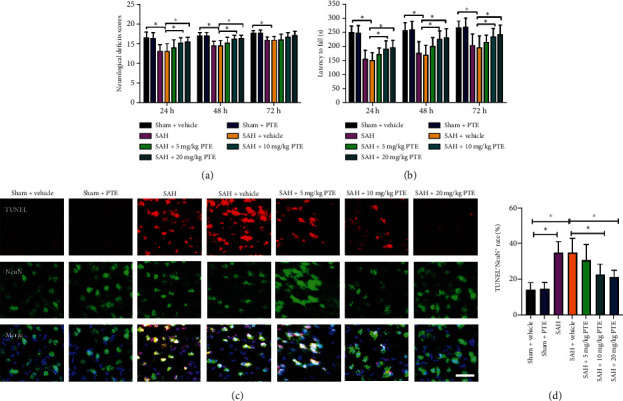
Influence of PTE on neurological outcomes and TUNEL apoptosis. PTE reduced deficits scores (a) and improved motor behavior (b) (*n* = 8 − 10/groups). Two-way ANOVA with Bonferroni post hoc test was employed. Representative microphotographs (c) and quantitative analyses (d) of the density of TUNEL^+^NEUN^+^ cells (*n* = 6/groups). One-way ANOVA with Bonferroni post hoc test was employed. Scale bar, 50 *μ*m. Error bars indicated the SD. ^∗^*P* < 0.05.

Inflammatory injury and oxidative insults could exacerbate neuronal death. SAH-induced neuronal death is closely associated with poor neurological deficits. As depicted in Figure 3, SAH aggravated neurological function (*P* < 0.05) and induced a high level of neuronal apoptosis (*P* < 0.001). By contrast, PTE treatment distinctly reduced the number of apoptotic neurons (*P* < 0.05) and improved the short-term neurological functions (*P* < 0.05) (Figures 3(a)–3(d)). These indicated that the cerebroprotective effects of PTE against EBI might partially be dependent on reduced cerebral inflammatory injury and oxidative insults.

### 3.4. PTE Treatment Induced SIRT1 Activation and Nrf2 Signaling Pathway

**Figure 4:**
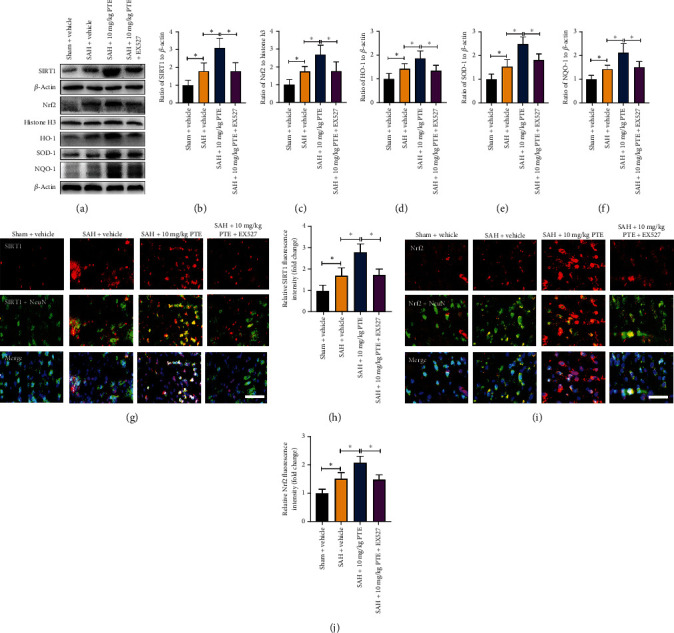
Influence of PTE on SIRT1-mediated Nrf2 cascade. Western blot images for SIRT1-mediated Nrf2 cascade (a). PTE markedly induced the expression of SIRT1 (b), nucleus Nrf2 (c), HO-1 (d), SOD-1 (e), and NQO-1 (f). In contrast, EX527 diminished the influence of PTE on SIRT1-mediated Nrf2 cascade (*n* = 6/groups). Immunofluorescence of SIRT1 (g) and Nrf2 staining (i). PTE markedly induced the expressions of SIRT1 (h) and nuclear Nrf2 in neurons (j), which could be abolished by EX527 (*n* = 6/groups). One-way ANOVA with Bonferroni post hoc test was employed. Scale bar, 50 *μ*m. Error bars indicated the SD. ^∗^*P* < 0.05.

PTE, an analog of RSV, has been verified as a potent SIRT1 activator. Meanwhile, accumulating evidence supports a critical role for SIRT1 in modulating Nrf2 activation. It revealed that PTE treatment markedly induced SIRT1 expression (*P* < 0.001) and nuclear distribution of Nrf2 (*P* = 0.0036) in the brain cortex (Figures 4(a)–4(c)). Moreover, PTE evidently enhanced the levels of HO-1, SOD-1, and NQO-1 (*P* = 0.03, *P* < 0.01, and *P* < 0.001, respectively) (Figures 4(d)–4(f)). Furthermore, SIRT1 and Nrf2 staining results indicated that PTE treatment significantly induced SIRT1 activation (*P* < 0.001) and Nrf2 translocation into nuclei (*P* < 0.001) (Figures 4(g)–4(j)). These suggested that PTE could activate SIRT1/Nrf2 signaling. In addition, we used EX527 to suppress SIRT1 activation. As depicted in Figure 4, EX527 prevented the increased SIRT1 expression and Nrf2 signaling pathway by PTE (*P* < 0.05) (Figures 4(a)–4(j)).

### 3.5. Inhibition of SIRT1 Abrogated PTE's Antioxidative Effects

**Figure 5:**
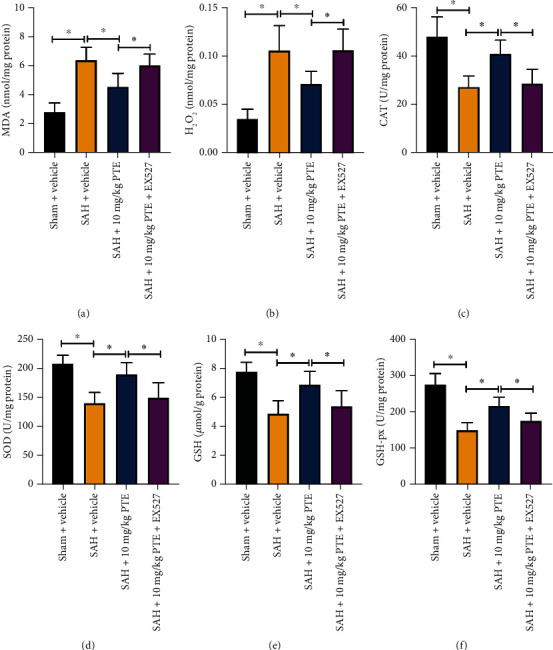
SIRT1 inhibition abated the anti-oxidant effects of PTE against SAH. Quantification of MDA (a), H_2_O_2_ (b), CAT (c), SOD (d), GSH (e), and GSH-Px (f) activities (*n* = 6/groups, one way ANOVA with Bonferroni post hoc test). Error bars indicated the SD. ^∗^*P* < 0.05.

Whether SIRT1 inhibition prevented PTE's antioxidant effects after SAH remains unknown. We observed that PTE provided a significant antioxidant property against SAH (*P* = 0.005 for MDA, *P* = 0.0247 for H_2_O_2_, *P* = 0.006 for CAT, *P* = 0.023 for SOD, *P* = 0.0057 for GSH, and *P* < 0.001 for GSH-px) (Figures 5(a)–5(f)). However, the effects of PTE on oxidative insults and the endogenous antioxidants were abolished by EX527 (*P* = 0.02 for MDA, *P* = 0.0239 for H_2_O_2_, *P* = 0.014 for CAT, *P* = 0.014 for SOD, *P* = 0.045 for GSH, and *P* = 0.0452 for GSH-px). EX527 markedly aggravated oxidative insults and further impaired the endogenous antioxidants after SAH (Figures 5(a)–5(f)). These results further indicated that the antioxidant property of PTE might partially be dependent on SIRT1 signaling.

### 3.6. SIRT1 Inhibition Reversed the Anti-Inflammatory Effects of PTE on SAH

**Figure 6:**
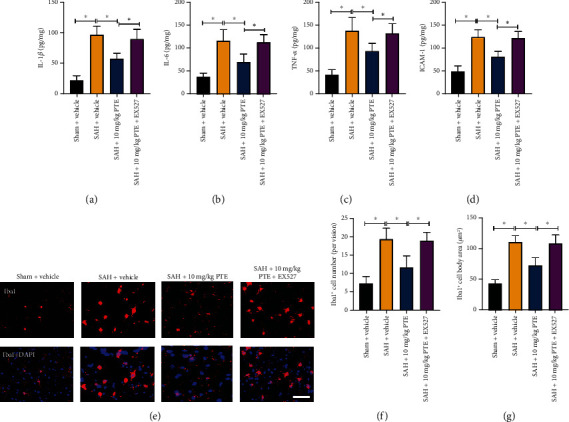
Suppression of SIRT1 abated the anti-inflammatory action of PTE. Quantitative analyses of IL-1*β* (a), IL-6 (b), TNF-*α* (c), and ICAM-1 (d) (*n* = 6/groups). Representative microphotographs (e) and quantitative analyses of the density of Iba1^+^ cells (f) and Iba^+^ cell body size (g) (*n* = 6/groups). One-way ANOVA with Bonferroni post hoc test was employed. Scale bar, 50 *μ*m. Error bars indicated the SD. ^∗^*P* < 0.05.

We further investigated the influence of SIRT1 inhibition on the anti-inflammatory effects of PTE. In congruent, it showed that PTE treatment significantly reduced proinflammatory cytokines release and microglia activation after SAH (*P* < 0.001 for IL-1*β*, *P* < 0.001 for IL-6, *P* = 0.0068 for TNF-*α*, and *P* < 0.001 for ICAM1). However, the anti-inflammatory effects by PTE were prevented by EX527 (*P* < 0.001 for IL-1*β*, *P* = 0.0022 for IL-6, *P* = 0.019 for TNF-*α*, and *P* < 0.001 for ICAM1) (Figures 6(a)–6(g)). EX527 further increased inflammatory mediators and activated microglia (*P* < 0.001) after SAH. These results indicated that the anti-inflammatory property of PTE might also partially be dependent on SIRT1 signaling.

### 3.7. SIRT1 Inhibition Reversed the Cerebroprotection of PTE on SAH

**Figure 7:**
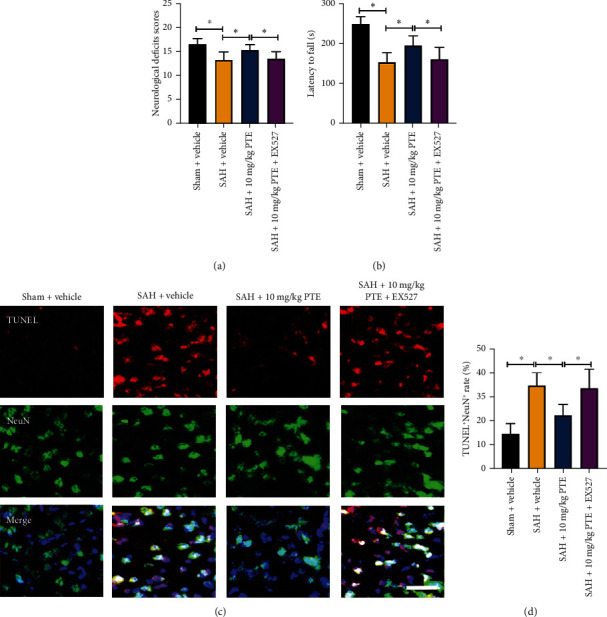
SIRT1 inhibition abated the beneficial actions of PTE on neurological scores and cellular apoptosis. Quantitative analyses of deficits scores (a) and rotarod function (b) (*n* = 10 − 12/groups). Representative microphotographs (c) and quantitative analyses (d) of TUNEL^+^NEUN^+^ cells in different groups (*n* = 6/groups). One-way ANOVA with Bonferroni post hoc test was employed. Scale bar, 50 *μ*m. Error bars indicated the SD. ^∗^*P* < 0.05.

Oxidative insults, inflammatory injury, and cell death are involved in EBI. Our data indicated that PTE treatment could reduce neuroinflammation, oxidative stress, and cell apoptosis and improve functional recovery after SAH. In addition, suppression of SIRT1 reversed the anti-inflammatory and antioxidative actions of PTE. We further evaluated the effects of SIRT1 inhibition on functional recovery and neuronal survival. Concomitant with the deteriorated oxidative injury and inflammatory impairment, EX527 abrogated the benefits of PTE on functional recovery (*P* = 0.0138 for neurological deficits score, and *P* = 0.0072 for motor function) and neuronal survival (*P* = 0.0127) (Figures 7(a)–7(d)).

## 4. Discussion

Inflammatory injury, oxidative insults, and cell apoptosis exert vital roles in SAH [2, 3, 21]. The present study revealed that PTE could significantly attenuate inflammatory response, oxidative damage, neuronal death, and early neurological deterioration after SAH. In addition, PTE induced SIRT1 activation and Nrf2 signaling pathway. EX527 pretreatment significantly diminished SIRT1-mediated Nrf2 signaling and abolished the neuroprotective actions of PTE following SAH. These data provide evidence that PTE protects against SAH insults by activating SIRT1-dependent Nrf2 signaling pathway.

PTE is a structural analog of RSV with higher bioavailability [16]. RSV is a well-known neuroprotective agent in different CNS diseases. In SAH area, RSV has been shown to ameliorate EBI and cerebral vasospasm and provide multifaceted neurovascular protection by inducing SIRT1-related signaling pathway [24–26]. In the meantime, RSV could significantly reduce oxidative damage and inflammatory insults after SAH [6, 26]. However, although research has also showed that PTE exhibits comprehensive protective actions, the exact role of PTE in SAH remains obscure. In central nervous system disorders, PTE is able to mitigate a variety of acute brain injuries, including traumatic brain injury (TBI), ischemic stroke, and SAH [16, 27]. Liu et al. reported that PTE reduced microglia-mediated neuroinflammation and neuronal oxidative damage in ischemic stroke [28]. Liu et al. suggested that PTE inhibited NLRP3 inflammasome and Nox2-mediated oxidative damage after SAH [16]. These previous studies indicated that PTE is a promising drug candidate for acute brain injuries. In congruent, the present study showed that PTE reduced the robust oxidative impairment and improved endogenous oxygen defensive enzymes. Moreover, PTE distinctly inhibited microglia activation and proinflammatory mediators after SAH. Oxidative damage and neuroinflammation directly result in cell apoptosis. Concomitant with the reduced oxidative insults and neuroinflammation, PTE reduced neuronal death and improved functional recovery after SAH.

Although a recent study has showed that PTE inhibited NLRP3 inflammasome and Nox2-signaling after SAH [16], large gaps in knowledge still exist regarding the mechanisms of PTE' s cerebroprotective effects. Nrf2 signaling has been implicated in EBI. Under normal circumstances, Nrf2 exerts a vital role in redox homeostasis and immune response. In addition, Nrf2 is fundamentally neuroprotective in other acute brain injuries, including cerebral ischemia, TBI, and intracerebral hemorrhage [15, 29–32]. Wang et al. reported that Nrf2 activation by melatonin receptor attenuated oxidative stress and neuroinflammation in a TBI mouse model [30]. Chen et al. demonstrated that Nrf2 activation by omaveloxolone inhibited M1-like microglia and ROS generation in intracerebral hemorrhage models [29]. Therefore, we evaluated changes in the Nrf2 signaling after PTE treatment. Our data revealed that PTE distinctly increased Nrf2 expression in nuclei as well as the subsequent pathways. However, it should be noted that Nrf2 plays a more important role in redox homeostasis. Recently, more and more studies indicated that Nrf2 also participated in modulating immune response by targeting different molecular targets. For example, Zhang et al. reported that Nrf2 activation could mediate HO-1 signaling to mitigate LPS-induced inflammation and lung injury [33]. Xiao et al. showed that Nrf2 could inhibit nod-like receptor pyrin domain-containing 3 inflammasome-mediated neuroinflammation and pyroptosis after cerebral ischemia-reperfusion injury [34]. In addition, Nrf2 has been demonstrated to regulate transforming growth factor-*β*, toll-like receptor 4/NF-кB, and Notch to affect microglial/macrophage polarization in different diseases models [35, 36]. However, whether PTE modulates these molecular targets after SAH remains obscure. Additional preclinical studies are still needed to clarify this. In summary, these data supported that Nrf2 signaling is involved in the neuroprotective effects of PTE.

SIRT1 was shown to implicate in different cellular biological functions [17, 21, 37, 38]. Evidence supports a critical role for SIRT1 in modulating Nrf2 activation in different diseases [39–42]. Zhang et al. reported that astaxanthin protected against TBI by modulating SIRT1/Nrf2/p38 signaling pathway [31]. Mao et al. indicated that si-SIRT1 abated SIRT1/Nrf2 activation by fucoxanthin in a model of renal ischemia-reperfusion injury [40]. PTE, a dimethylated analog of RSV, has also been verified as a potent SIRT1 activator in different research fields [43–45]. However, whether PTE mediates SIRT1 signaling in experimental SAH remains unknown. We further used a SIRT1 selective inhibitor EX527 to investigate the interaction between SIRT1 and Nrf2. In this study, EX527 distinctly suppressed SIRT1 and Nrf2 expressions and subsequent molecular targets after SAH. Moreover, we observed that EX527 pretreatment deteriorated the cerebroprotection of PTE against SAH. Overall, these data indicated that the neuroprotection conferred by PTE after SAH was through SIRT1-dependent Nrf2 signaling.

Our study has several limitations. First, whether PTE administration could improve cognitive function and reduce the delayed cerebral ischemia after SAH remain unknown. Second, we did not evaluate the possible beneficial effects of PTE on female rats. Third, Nrf2 was shown to positively mediate SIRT1 signaling in other diseases [46]. The exact interaction between SIRT1 and Nrf2 signaling in experimental SAH still needs to be clearly elucidated.

## 5. Conclusion

In conclusion, we revealed that PTE protected against brain damage after SAH via reducing neuroinflammatory injury, oxidative insults, and cellular apoptosis. By interacting with SIRT1/Nrf2 signaling, PTE attenuated EBI and improved neurological outcomes after SAH.

## Data Availability

The data can be directed to the corresponding author upon reasonable request.

## Supplementary Materials

Supplementary Materials Supplementary Table 1: animals groups and mortality rates.

## References

[1] Macdonald R. L., Schweizer T. A. (2017). Spontaneous subarachnoid haemorrhage. *Lancet*.

[2] Lu Y., Zhang X. S., Zhou X. M. (2019). Peroxiredoxin 1/2 protects brain against H2O2-induced apoptosis after subarachnoid hemorrhage. *The FASEB Journal*.

[3] Liu G. J., Tao T., Zhang X. S. (2021). Resolvin D1 attenuates innate immune reactions in experimental subarachnoid hemorrhage rat model. *Molecular Neurobiology*.

[4] Heinz R., Brandenburg S., Nieminen-Kelha M. (2021). Microglia as target for anti-inflammatory approaches to prevent secondary brain injury after subarachnoid hemorrhage (SAH). *Journal of Neuroinflammation*.

[5] Zhou X. M., Zhou M. L., Zhang X. S. (2014). Resveratrol prevents neuronal apoptosis in an early brain injury model. *The Journal of Surgical Research*.

[6] Zhang X. S., Li W., Wu Q. (2016). Resveratrol attenuates acute inflammatory injury in experimental subarachnoid hemorrhage in rats via inhibition of TLR4 pathway. *International Journal of Molecular Sciences*.

[7] Ma C., Xiang J., Huang G. (2021). Pterostilbene alleviates cholestasis by promoting SIRT1 activity in hepatocytes and macrophages. *Frontiers in Pharmacology*.

[8] Yashiro T., Yura S., Tobita A., Toyoda Y., Kasakura K., Nishiyama C. (2020). Pterostilbene reduces colonic inflammation by suppressing dendritic cell activation and promoting regulatory T cell development. *The FASEB Journal*.

[9] Beghelli D., Zallocco L., Barbalace M. C. (2022). Pterostilbene Promotes Mean Lifespan in Both Male and Female *Drosophila Melanogaster* Modulating Different Proteins in the Two Sexes. *Oxidative Medicine and Cellular Longevity*.

[10] Yu C. L., Yang S. F., Hung T. W., Lin C. L., Hsieh Y. H., Chiou H. L. (2019). Inhibition of eIF2*α* dephosphorylation accelerates pterostilbene-induced cell death in human hepatocellular carcinoma cells in an ER stress and autophagy- dependent manner. *Cell Death & Disease*.

[11] Lacerda D., Ortiz V., Turck P. (2018). Stilbenoid pterostilbene complexed with cyclodextrin preserves left ventricular function after myocardial infarction in rats: possible involvement of thiol proteins and modulation of phosphorylated GSK-3*β*. *Free Radical Research*.

[12] Curtis M. J., Alexander S., Cirino G. (2018). Experimental design and analysis and their reporting II: updated and simplified guidance for authors and peer reviewers. *British Journal of Pharmacology*.

[13] McGrath J. C., Lilley E. (2015). Implementing guidelines on reporting research using animals (ARRIVE etc.): new requirements for publication in BJP. *British Journal of Pharmacology*.

[14] Zhang X. H., Peng L., Zhang J. (2020). Berberine ameliorates subarachnoid hemorrhage injury via induction of sirtuin 1 and inhibiting HMGB1/Nf-*κ*B pathway. *Frontiers in Pharmacology*.

[15] Zhang Z. H., Liu J. Q., Hu C. D. (2021). Luteolin confers cerebroprotection after subarachnoid hemorrhage by suppression of NLPR3 inflammasome activation through Nrf2-dependent pathway. *Oxidative Medicine and Cellular Longevity*.

[16] Liu H., Zhao L., Yue L. (2017). Pterostilbene attenuates early brain injury following subarachnoid hemorrhage via inhibition of the NLRP3 inflammasome and Nox2-related oxidative stress. *Molecular Neurobiology*.

[17] Xia D. Y., Yuan J. L., Jiang X. C. (2021). SIRT1 promotes M2 microglia polarization via reducing ROS-mediated NLRP3 inflammasome signaling after subarachnoid hemorrhage. *Frontiers in Immunology*.

[18] Sugawara T., Ayer R., Jadhav V., Zhang J. H. (2008). A new grading system evaluating bleeding scale in filament perforation subarachnoid hemorrhage rat model. *Journal of Neuroscience Methods*.

[19] Sreenivasmurthy S. G., Iyaswamy A., Krishnamoorthi S. (2022). Protopine promotes the proteasomal degradation of pathological tau in Alzheimer's disease models via HDAC6 inhibition. *Phytomedicine*.

[20] Iyaswamy A., Krishnamoorthi S. K., Zhang H. (2021). Qingyangshen mitigates amyloid-*β* and Tau aggregate defects involving PPAR*α*- TFEB activation in transgenic mice of Alzheimer's disease. *Phytomedicine*.

[21] Zhang X. S., Lu Y., Li W. (2021). Cerebroprotection by dioscin after experimental subarachnoid haemorrhage via inhibiting NLRP3 inflammasome through SIRT1-dependent pathway. *British Journal of Pharmacology*.

[22] Xu X., Gao W., Cheng S. (2017). Anti-inflammatory and immunomodulatory mechanisms of atorvastatin in a murine model of traumatic brain injury. *Journal of Neuroinflammation*.

[23] Yuan B., Zhou X. M., You Z. Q. (2020). Inhibition of AIM2 inflammasome activation alleviates GSDMD-induced pyroptosis in early brain injury after subarachnoid haemorrhage. *Cell Death & Disease*.

[24] Clarke J. V., Brier L. M., Rahn R. M. (2022). SIRT1 mediates hypoxic postconditioning- and resveratrol-induced protection against functional connectivity deficits after subarachnoid hemorrhage. *Journal of Cerebral Blood Flow and Metabolism*.

[25] Diwan D., Vellimana A. K., Aum D. J. (2021). Sirtuin 1 mediates protection against delayed cerebral ischemia in subarachnoid hemorrhage in response to hypoxic postconditioning. *Journal of the American Heart Association*.

[26] Zhang X., Wu Q., Zhang Q. (2017). Resveratrol attenuates early brain injury after experimental subarachnoid hemorrhage via inhibition of NLRP3 inflammasome activation. *Frontiers in Neuroscience*.

[27] Yang Y., Wang J., Li Y. (2016). HO-1 signaling activation by pterostilbene treatment attenuates mitochondrial oxidative damage induced by cerebral ischemia reperfusion injury. *Molecular Neurobiology*.

[28] Liu H., Wu X., Luo J. (2019). Pterostilbene attenuates astrocytic inflammation and neuronal oxidative injury after ischemia-reperfusion by inhibiting NF-*κ*B phosphorylation. *Frontiers in Immunology*.

[29] Hu L., Cao Y., Chen H. (2022). The novel Nrf2 activator omaveloxolone regulates microglia phenotype and ameliorates secondary brain injury after intracerebral hemorrhage in mice. *Oxidative Medicine and Cellular Longevity*.

[30] Wang J., Jiang C., Zhang K. (2019). Melatonin receptor activation provides cerebral protection after traumatic brain injury by mitigating oxidative stress and inflammation via the Nrf2 signaling pathway. *Free Radical Biology & Medicine*.

[31] Zhang X. S., Lu Y., Li W. (2021). Astaxanthin ameliorates oxidative stress and neuronal apoptosis via SIRT1/NRF2/Prx2/ASK1/p38 after traumatic brain injury in mice. *British Journal of Pharmacology*.

[32] Wang H., Zhou X. M., Wu L. Y. (2020). Aucubin alleviates oxidative stress and inflammation via Nrf2-mediated signaling activity in experimental traumatic brain injury. *Journal of Neuroinflammation*.

[33] Zhang Y., Yang S., Qiu Z. (2022). Pyrogallol enhances therapeutic effect of human umbilical cord mesenchymal stem cells against LPS-mediated inflammation and lung injury via activation of Nrf2/HO-1 signaling. *Free Radical Biology & Medicine*.

[34] Xiao L., Dai Z., Tang W., Liu C., Tang B. (2021). Astragaloside IV alleviates cerebral ischemia-reperfusion injury through NLRP3 inflammasome-mediated pyroptosis inhibition via activating Nrf2. *Oxidative Medicine and Cellular Longevity*.

[35] Wang L., He C. (2022). Nrf2-mediated anti-inflammatory polarization of macrophages as therapeutic targets for osteoarthritis. *Frontiers in Immunology*.

[36] Arfmann-Knubel S., Struck B., Genrich G. (2015). The crosstalk between Nrf2 and TGF-*β*1 in the epithelial-mesenchymal transition of pancreatic duct epithelial cells. *PLoS One*.

[37] Zhang J., Bi R., Meng Q. (2019). Catalpol alleviates adriamycin-induced nephropathy by activating the SIRT1 signalling pathway in vivo and in vitro. *British Journal of Pharmacology*.

[38] Wu B., Feng J. Y., Yu L. M. (2018). Icariin protects cardiomyocytes against ischaemia/reperfusion injury by attenuating sirtuin 1-dependent mitochondrial oxidative damage. *British Journal of Pharmacology*.

[39] Zhang X., Wu Q., Lu Y. (2018). Cerebroprotection by salvianolic acid B after experimental subarachnoid hemorrhage occurs via Nrf2- and SIRT1-dependent pathways. *Free Radical Biology & Medicine*.

[40] Mao H., Wang L., Xiong Y., Jiang G., Liu X. (2022). Fucoxanthin attenuates oxidative damage by activating the Sirt1/Nrf2/HO-1 signaling pathway to protect the kidney from ischemia-reperfusion injury. *Oxidative Medicine and Cellular Longevity*.

[41] Huang X., Shi Y., Chen H. (2020). Isoliquiritigenin prevents hyperglycemia-induced renal injuries by inhibiting inflammation and oxidative stress via SIRT1-dependent mechanism. *Cell Death & Disease*.

[42] Ma W., Guo W., Shang F. (2020). Bakuchiol alleviates hyperglycemia-induced diabetic cardiomyopathy by reducing myocardial oxidative stress via activating the SIRT1/Nrf2 signaling pathway. *Oxidative Medicine and Cellular Longevity*.

[43] Zhu Q., Tang T., Liu H. (2020). Pterostilbene attenuates cocultured BV-2 microglial inflammation-mediated SH-SY5Y neuronal oxidative injury via SIRT-1 signalling. *Oxidative Medicine and Cellular Longevity*.

[44] Song L., Chen T. Y., Zhao X. J. (2019). Pterostilbene prevents hepatocyte epithelial-mesenchymal transition in fructose-induced liver fibrosis through suppressing miR-34a/Sirt1/p53 and TGF-*β*1/Smads signalling. *British Journal of Pharmacology*.

[45] Chen Y., Zhang H., Ji S. (2021). Resveratrol and its derivative pterostilbene attenuate oxidative stress- induced intestinal injury by improving mitochondrial redox homeostasis and function via SIRT1 signaling. *Free Radical Biology & Medicine*.

[46] Huang K., Gao X., Wei W. (2017). The crosstalk between Sirt1 and Keap1/Nrf2/ARE anti-oxidative pathway forms a positive feedback loop to inhibit FN and TGF-*β*1 expressions in rat glomerular mesangial cells. *Experimental Cell Research*.

