# Supplementary material for: Pterostilbene Attenuates Subarachnoid Hemorrhage-Induced Brain Injury through the SIRT1-Dependent Nrf2 Signaling Pathway
Source: Oxid Med Cell Longev. 2022 Nov 30;2022:3550204. doi: 10.1155/2022/3550204 (PMC9729048; doi:10.1155/2022/3550204)
Supplement: Supplementary Materials — Supplementary Table 1: animals groups and mortality rates. [file 3550204.f1.docx]

Supplementary Table 1. Animals groups and Mortality rates

| Groups | Alive | Died | Mortality rates |
| --- | --- | --- | --- |
| sham + vehicle | 24 | 0 | 0 |
| sham + 10 mg/kg PTE group | 12 | 0 | 0 |
| SAH | 12 | 2 | 14.3% |
| SAH + vehicle | 24 | 6 | 20% |
| SAH + 5 mg/kg PTE group | 12 | 3 | 20% |
| SAH + 10 mg/kg PTE group | 24 | 3 | 11.1% |
| SAH + 20 mg/kg PTE group | 12 | 2 | 14.3% |
| SAH + 10 mg/kg PTE + EX527 | 12 | 3 | 20% |
